# Microstructure and Mechanical Behavior of Porous Ti–6Al–4V Processed by Spherical Powder Sintering

**DOI:** 10.3390/ma6104868

**Published:** 2013-10-23

**Authors:** Lucía Reig, Concepción Tojal, David J. Busquets, Vicente Amigó

**Affiliations:** 1 Department of Mechanical Engineering and Construction, Universitat Jaume I, Av. Sos Baynat s/n, 12071 Castelló de la Plana, Spain; 2 Materials Technology Institute, Universitat Politècnica de València, Camino de Vera s/n, 46022 Valencia, Spain; E-Mails: contodo@upvnet.upv.es (C.T.); dbusquets@mcm.upv.es (D.J.B.); vamigo@mcm.upv.es (V.A.)

**Keywords:** porous titanium, microsphere sintering, bending strength, compressive strength, stiffness, metallic implant

## Abstract

Reducing the stiffness of titanium is an important issue to improve the behavior of this material when working together with bone, which can be achieved by generating a porous structure. The aim of this research was to analyze the porosity and mechanical behavior of Ti–6Al–4V porous samples developed by spherical powder sintering. Four different microsphere sizes were sintered at temperatures ranging from 1300 to 1400 °C for 2, 4 and 8 h. An open, interconnected porosity was obtained, with mean pore sizes ranging from 54.6 to 140 µm. The stiffness of the samples diminished by as much as 40% when compared to that of solid material and the mechanical properties were affected mainly by powder particles size. Bending strengths ranging from 48 to 320 MPa and compressive strengths from 51 to 255 MPa were obtained.

## 1. Introduction

Increased use of titanium and its alloys as biomaterials stems from their lightness, biocompatibility, corrosion resistance and excellent mechanical properties when compared to more conventional stainless steel and cobalt-based alloys [1]. Conversely, their excessive stiffness in comparison to that of cortical human bone (0.16–18 GPa [2]), relatively high cost and reactivity have been reported to be major disadvantages [3,4,5,6].

As explained by Sumitomo *et al.* [5] and Niinomi [6], the major differences between the stiffness of the bone and that of the implant may alter the bone tissue reaction at the interface between both elements, leading to local loss of osseous material. The scientific community has been seeking alternatives to mitigate this problem. Several works [1,7,8,9,10], have focused on developing low modulus β-type titanium alloys, most of which are composed of non-toxic elements. Examples of these alloys include Ti–29Nb–13Ta–4.6Zr (referred to as TNTZ, 60 GPa) [1], Ti–12Mo–6Zr–2Fe (referred to as TMZF, 74 to 86 GPa) [7], Ti25Ta (64 GPa) [8], Ti–35Nb–6Ta (50 to 75 GPa) [9] or Ti20Nb (74 GPa) [10]. Stiffness has also been reduced by creating a porous structure. Among the different methods used, Nugroho *et al.* [11] successfully combined hot isostatic pressing (HIP) and the pressurized gas bubble entrapment method to produce porous titanium samples with up to 45 vol % of porosity (20–200 μm). In the work by Barbas *et al.* [12], the selective laser melting (SLM) method was used to develop Commercially Pure Titanium samples with 53 vol % porosity and pore sizes falling within the 860–1500 µm range. Wieding *et al.* [13] also used SLM to fabricate Ti–6Al–4V scaffolds with porosities of approximately 70 vol %, and compressive strengths within the human cortical bone range. Highly porous scaffolds were also obtained by the space holder method. While Dezfuli *et al.* [14] observed excellent attachment and proliferation of human cells to scaffolds fabricated with urea, in the studies by Reig *et al.* [15,16], of all the parameters influencing the mechanical properties of the porous samples developed, the amount and size of spacer particles, together with the compacting pressure, proved to be the most essential.

Titanium microsphere sintering, which has traditionally been used to coat titanium with a view to improving cell attachment [17], also successfully obtained completely porous structures. The metallurgical process followed to develop porous Ti–6Al–4V samples by microsphere sintering was optimized in [4]. While the reactivity observed when sintering in alumina molds increased the brittleness of samples and diminished their mechanical properties, minimal reactivity occurred when molds were covered with an yttria layer. Furthermore, the study concluded that a compromise between mechanical properties (better for the smallest microspheres) and pore size (greater for the coarser microspheres) should be reached for biomedical applications. However, only two different particle sizes were sintered and no data on the material’s compressive behavior were supplied in that study.

In this paper, the porosity and mechanical behavior (bending strength, compressive strength and stiffness) of porous samples processed by sintering spherical powder of four different sizes have been analyzed. For the development of these samples and in order to make them appropriate for biomedical applications, the following criteria were taken into consideration:

(1) Minimal stiffness when compared to that of solid Ti–6Al–4V titanium alloy.
(2) Achievement of a strength equivalent (or higher) to that of cortical or cancellous human bone tissue.
(3) Open porosity and interconnected pores.
(4) Porosity in the range of 20% to 80%, as defined by Bansiddhi *et al.* [18].
(5) Pore size greater than 100 µm to facilitate bone colonization [19].

Furthermore, machining the samples after the sintering process results critical, due to beads tend to come off the sample when they are subjected to cutting or grinding, especially as the microsphere size increases. So, in order to assess the reactivity of titanium with the mold, and determine whether machining could be avoided after the sintering process, no machining was applied after the sintering process.

## 2. Experimental Procedure

### 2.1. Raw Material

**Figure 1:**
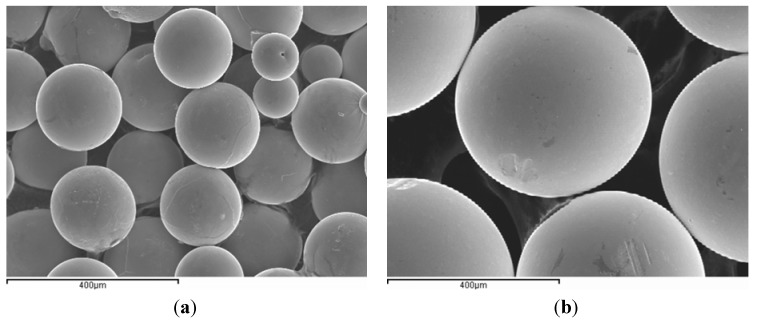
Morphology of the Ti–6Al–4V spherical powder produced by the plasma-rotating electrode process (PREP): (**a**) Fine particles (−250/+180 µm); (**b**) Coarse particles (−600/+425 µm).

**Figure 2:**
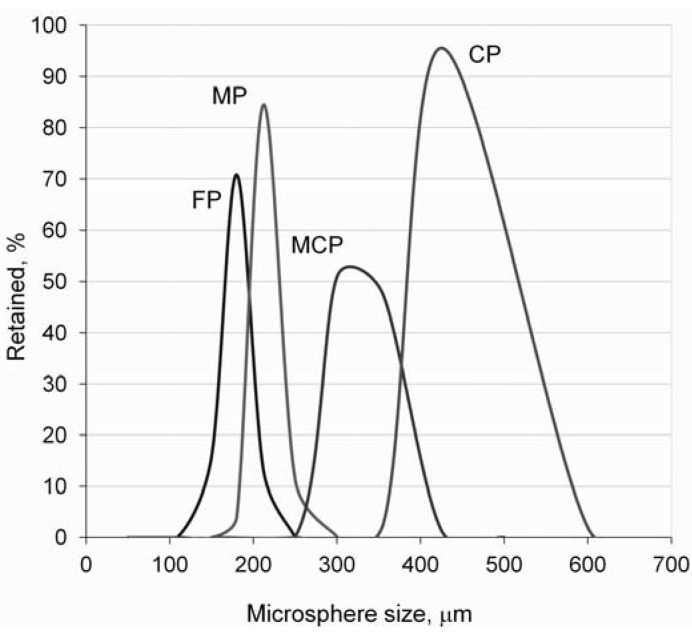
Particle size distribution of the Ti–6Al–4V microspheres.

Ti–6Al–4V alloy spherical powder (Figure 1), manufactured by the plasma-rotating electrode process (PREP), was supplied by Phelly Materials Inc. As shown in Figure 2, four different particle sizes were selected for this study. Although the particle size of selected particles (+180/−600 µm) is relatively large in comparison to much smaller titanium particles (*i.e*., that used for the laser or electron beam melting process), to make it clear and simple to understand, they were referenced as “fine” (FP), “medium” (MP), “medium-coarse” (MCP) and “coarse” (CP) in the present paper. Their chemical composition agreed with the specification for Ti–6Al–4V powders used for coating surgical and medical implants (ASTM F1580-01), and they all had an aluminum content of between 5.5 and 6.75 wt % and a Vanadium content below 4.5 wt %. The amount of hydrogen, nitrogen and oxygen was under 0.004 wt %, 0.03 wt % and 0.20 wt %, respectively, denoting high purity powder.

The density of the raw material was determined according to Standard ASTM B213-97. Although apparent density was lower for the smaller microspheres (FP: 2.64 g/cm^3^, MP: 2.63 g/cm^3^, MCP: 2.74 g/cm^3^, CP: 2.82 g/cm^3^), after applying slight vibration (tap density), it increased up to 2.82 g/cm^3^ in them all, irrespectively of their mean diameter in each granulometric distribution.

### 2.2. Equipment and Methods

Yttria-coated alumina molds were used for the sintering process. The microspheres sizes selected for the present study, and the sintering temperatures and times are based on previous research [4], and are summarized in Table 1. After pouring the Ti–6Al–4V microspheres into the mold, a slight vibration was applied to increase the number of contacts between microspheres. Sintering was performed in an HVT 15/75/450 Carbolite vacuum furnace (10^−4^–10^−5^ mBar).

**Table 1:** Spherical powder sintering process variables.

Process variables	Values	Unit
Sintering temperatures	1300, 1350, 1400	°C
Sintering times	2, 4, 8	h
Microsphere sizes	FP: −250/+180	µm
	MP: −300/+212	
	MCP: −425/+300	
	CP: −600/+425	

### 2.3. Characterization of the Porous Samples

A microstructural analysis was performed by optical and electron microscopy in a Nikon Microphot FX and a JEOL JSM6300 Scanning Electron microscope, respectively. The apparent density and porosity of the developed samples was analyzed by mercury intrusion porosimetry (MIP, AutoPore IV 9500) using pressures from 2 psi (13.8 kPa) to 32989 psi (227.4 MPa), equivalent to pores with diameters ranging from 91.2 µm to 5.5 nm. Pressures were converted to equivalent pore widths using the Washburn equation, assuming a contact angle of 130°. Five rectangular specimens (≈ 4 ×·10.5·× 25 mm^3^) were subjected to the three-point bending test, which was performed according to ASTM E290-97a specifications in an Instron 4204, at a crosshead speed of 0.5 mm/min. Intermediate microsphere sizes (MP and MCP) were selected for the compressive strength test as they were expected to provide a compromise between strength and pore size. Five cylindrical samples (ϕ = 14 mm; h ≈ 23 mm) were tested under compression, in accordance with ASTM E9-89a specifications (0.2 mm/min). Stiffness was determined in compression by following the ASTM E111-97 standard test method. The stiffness of rod forged Ti–6Al–4V was used as a reference to evaluate the relative stiffness of the developed porous samples (*E*_cr_ = *E*_c_/*E*_cs_, where *E*_c_ is the stiffness of each porous sample and *E*_cs_ is that of solid material).

## 3. Results

### 3.1. Microstructure

**Figure 3:**
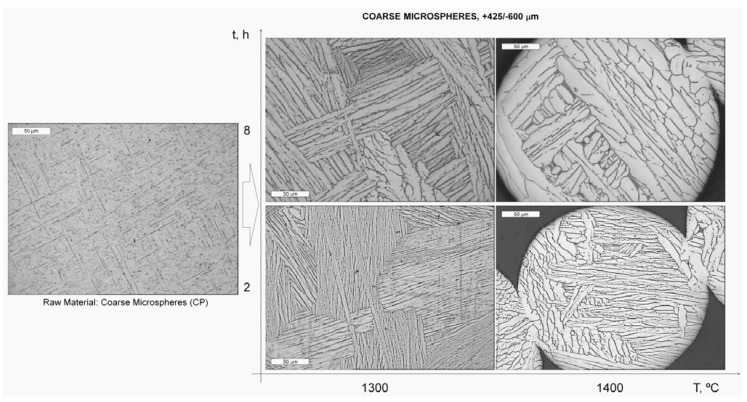
Microstructure of the Ti–6Al–4V microspheres (CP) before and after the sintering process. Etching: Kroll reagent.

Figure 3 presents the microstructure of the spherical particles before and after the sintering process. Before sintering, alternating α and β thin layers (Widmanstätten) are distinguished which, according to Leyens and Peters [20], typically originates from the high cooling rate of the PREP process. As expected, microstructure coarsening occurred when sintering, and the increased size of the grains was greater when sintering at higher temperatures or for longer times.

### 3.2. Porosity

**Figure 4:**
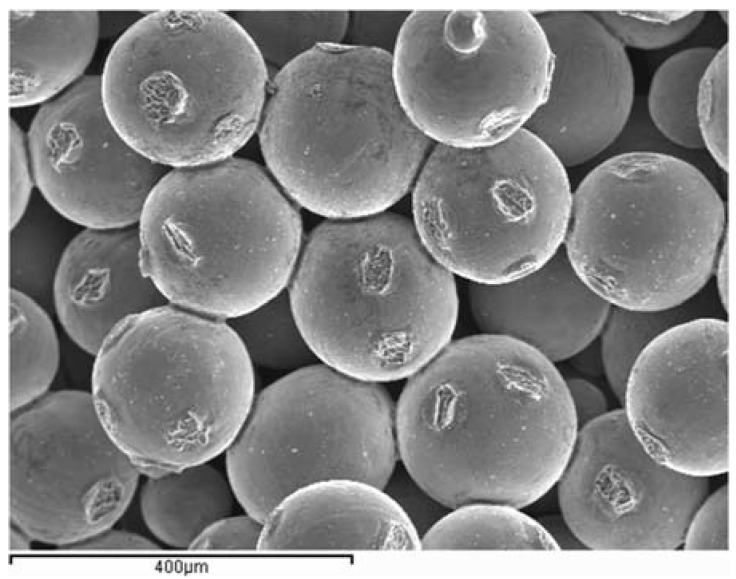
SEM micrograph of the FP Ti–6Al–4V microspheres after being sintered at 1300 °C for 8 h. Bending test fracture area.

The mercury porosimetry test results are summarized in Table 2. A very similar porosity (≈31.5 vol %) and density (≈2.95 g/cm^3^) were found for all the samples, regardless of their particle size. These values are close to that presented by theoretical body-centered cubic structures (BCC), with a packing factor of 0.68 (32% porosity). The little differences observed when sintering different microspheres sizes, as well as the higher porosity obtained when compared with denser close-packed structures (FCC, 26% porosity), are attributed to the particle-size distribution of the microspheres and some degree of random arrangement into the mold. The slight increase in density when compared to the tap density of the raw material (≈2.82 g/cm^3^) was attributed to the developed sintering necks between adjacent microspheres during the sintering process (see Figure 4), which slightly shrinked the original volume. As expected, larger powder particles resulted in larger pore sizes.

**Table 2:** Porosimetry test results.

Sample	Apparent density g/cm^3^	Median diameter µm	d84 * vol %	Open porosity Vol %
FP	2.95	54.6	72.7	32.1
MP	2.95	76.6	97.2	31.7
MCP	2.94	113.4	161.3	32.5
CP	2.95	140.1	175.9	31.2

* 84 vol % of pores below this size

### 3.3. Mechanical Properties

#### 3.3.1. Bending Strength

**Figure 5:**
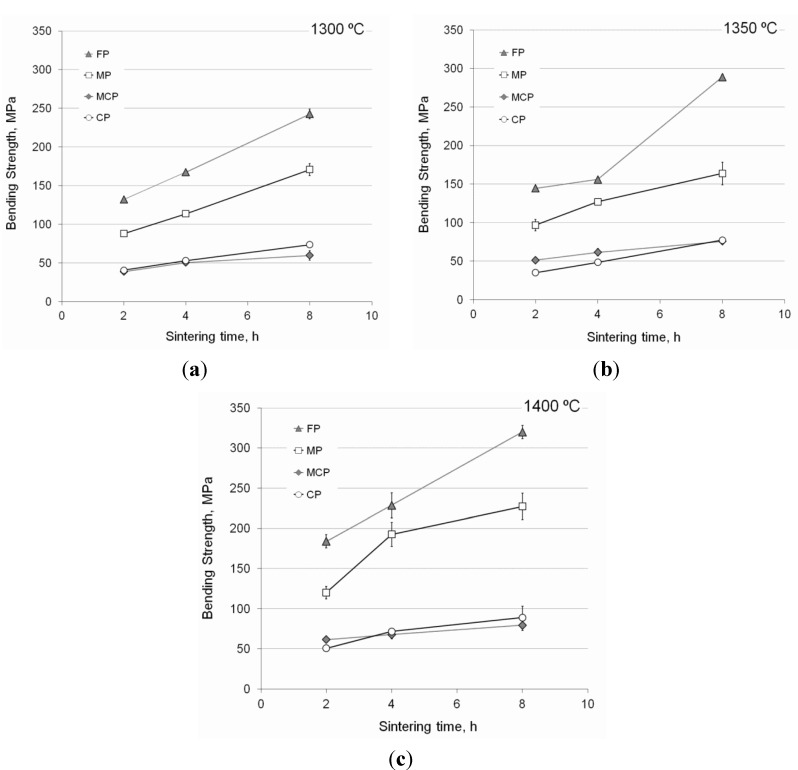
Bending strength and standard deviation of the porous samples developed by sintering Ti–6Al–4V microspheres: (**a**) 1300 °C; (**b**) 1350 °C; (**c**) 1400 °C.

Flexural strength and the standard deviation of the porous samples developed by Ti–6Al–4V spherical powder sintering are summarized in Figure 5. Although better mechanical properties were obtained when raising the temperature and prolonging the sintering time, particle size proved to be the most influential parameter, giving rise to higher bending strength values when sintering smaller microspheres. According to German [21], this is due to their larger specific surface, which provides more energy to develop necks between neighboring microspheres in the sintering process. Although larger differences were expected between MCP and CP porous samples, similar results were obtained for all the sintering cycles applied.

#### 3.3.2. Compressive Strength

**Figure 6:**
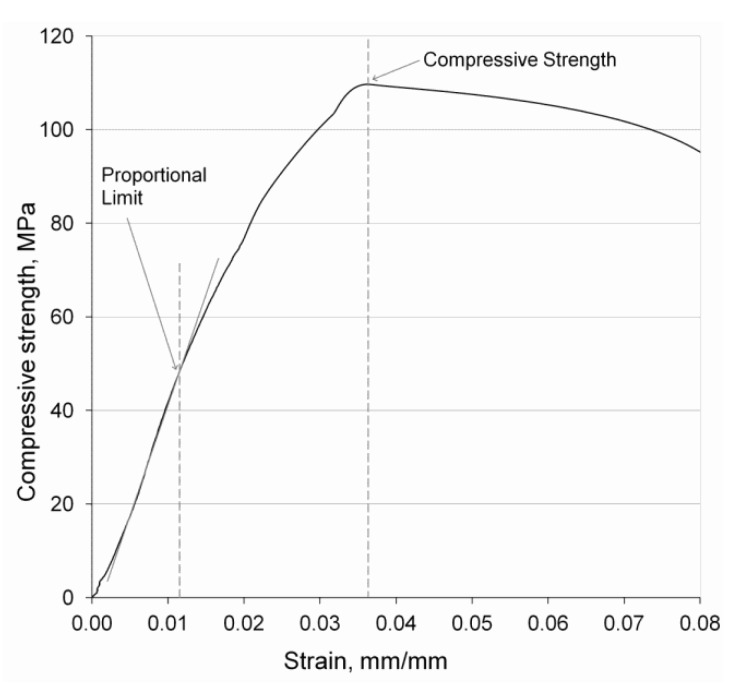
Stress-strain diagram of the MP Ti–6Al–4V microspheres sintered at 1400 °C for 2 h.

The generic stress-strain compressive curve of the developed porous samples is plotted in Figure 6. The stiffness of the material was determined as the stress/strain ratio at the proportional limit, while the compressive strength was considered the maximum stress sustainable under crush loading.

**Figure 7:**
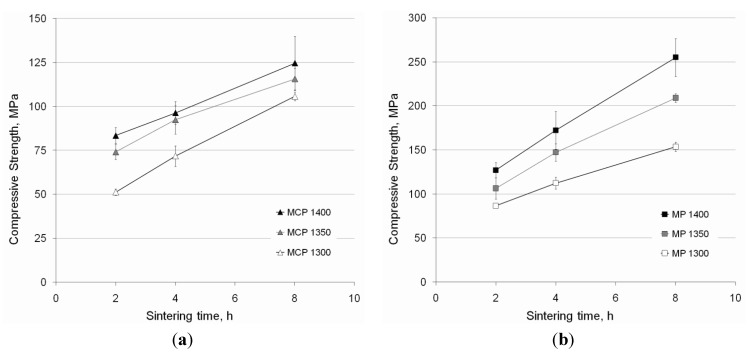
Compressive strength and standard deviation of the porous samples developed by sintering Ti–6Al–4V microspheres: (**a**) MCP (−425/+300 µm); (**b**) MP (−300/+212 µm).

Figure 7 summarizes the compressive strength and the standard deviation of the sintered MCP and MP particles. Similarly to bending strength, better compressive behavior was presented by smaller particles, and it improved with higher temperatures and longer sintering times. The compressive strength values ranged from 51 to 125 MPa, and from 86 to 255 MPa for the MCP and MP porous samples, respectively. The compressive strength of the MCP microspheres sintered at 1400 °C for 8 h was slightly lower than expected. This was attributed to reactivity with the alumina of the substrate through the yttria coating [4], which was most probably not uniform or thick enough.

#### 3.3.3. Stiffness

**Figure 8:**
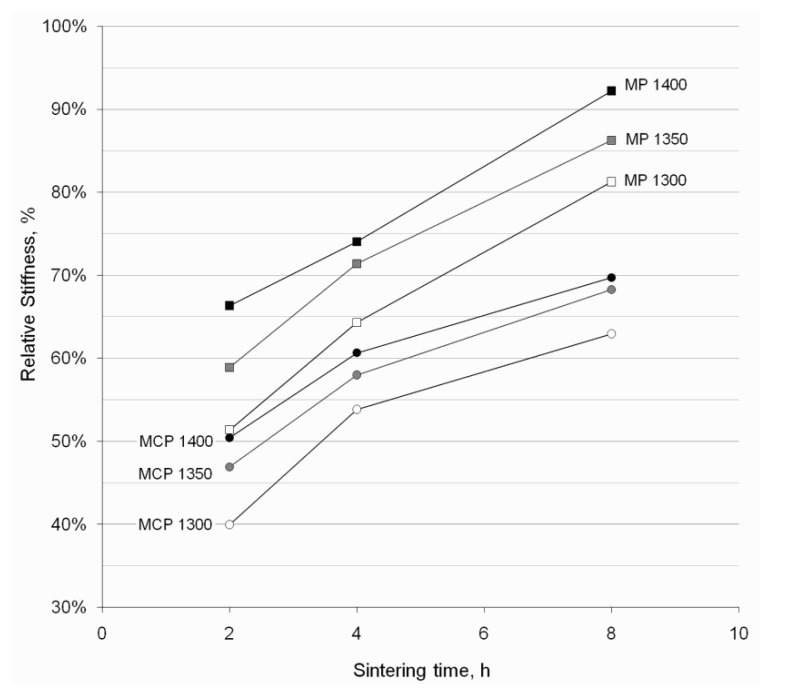
Relative stiffness of the porous Ti–6Al–4V samples developed by microsphere sintering.

The stiffness of the porous developed samples, in relation to rod forged Ti–6Al–4V, is shown in Figure 8. Similarly to strength, higher stiffness values were obtained when sintering smaller particles (MP), and they generally increased when sintering at higher temperatures or for longer times. The stiffness of the developed porous samples ranged from 40% to 92% of that of solid material which, as reported by Ryan *et al.* [22], is of vital importance to minimize bone re-absorption problems.

## 4. Discussion

Porous Ti–6Al–4V samples with an open and interconnected porosity, and a median pore diameter ranging from 54.6 to 140.1 µm, were obtained by spherical powder sintering. According to Bansiddhi *et al.* [18], a minimum pore size of 500 µm is required to allow the generation of blood vessels, whereas authors such as Bram *et al.* [19] reported that one of at least 100 µm is desirable to generate bone cells. However in the study by Kujala *et al.* [23], bone in-growth was also observed with smaller pore sizes (50 to 125 µm) when no load was applied. Thus, despite larger pore sizes (CP and MCP porous samples) facilitating bone in-growth, vascularization can also be expected in the FP and MP porous specimens when load is lacking.

Compressive strength values within the 130–205 MPa range were reported in [2,24] for cortical human bone, which were lower for cancellous bone tissue (≈70 MPa for femoral head; ≈10 MPa for vertebrae). Furthermore, a bending strength close to 100 MPa was observed by Heimann [25] for the cortical human bone. So, although CP and MCP porous samples could be considered to replace some cancellous bones, their strength is not sufficient to allow their use as a cortical bone substitute. Conversely, although the samples obtained after sintering the smallest particles (FP) presented the best mechanical properties, bone in-growth possibilities greatly reduced, thus rendering their use unsuitable. In contrast, the samples developed from sintered MP spherical powder showed slightly larger pore sizes, together with appropriate mechanical properties (both bending and compressive strengths ranging from approximately 90 to 225 MPa, and relative stiffness from 50% to 88%), which make them good candidates to replace cancellous and some cortical human bones. So, while porous samples produced by CP and MCP spherical powder sintering would be the most appropriate to replace cancellous bone tissue, MP microsphere sintering would be the most recommendable option to develop implant devices replacing failed hard tissue (*i.e*., titanium intervertebral discs or bone plates).

## 5. Conclusions

Porous Ti–6Al–4V has been developed by spherical powder sintering to provide a new material which responds to medical problem necessities. Samples with approximately 30 vol % open porosity, and a wide range of compressive and bending strength values, were obtained when sintering four different granulometric fractions in different temperature-time cycles. The stiffness of the developed porous material varied from 40% to 88% of that of solid Ti–6Al–4V. While MCP porous samples can be considered candidates to substitute some cancellous bones, the properties of sintered MP specimens make them good candidates for both cortical and cancellous bone applications.
